# Effectiveness of virtual and augmented reality for cardiopulmonary resuscitation training: a systematic review and meta-analysis

**DOI:** 10.1186/s12909-024-05720-8

**Published:** 2024-07-05

**Authors:** Rao Sun, Yixuan Wang, Qingya Wu, Shuo Wang, Xuan Liu, Pei Wang, Yuqin He, Hua Zheng

**Affiliations:** 1 Department of Anesthesiology and Pain Medicine, Hubei Key Laboratory of Geriatric Anesthesia and Perioperative Brain Health, and Wuhan Clinical Research Center for Geriatric Anesthesia, Tongji Hospital, Tongji Medical College, Huazhong University of Science and Technology, Wuhan, China; 2 School of Public Administration, Hubei University, Wuhan, China; 3 Department of Anesthesiology, Shanxi Bethune Hospital, Shanxi Academy of Medical Sciences, Third Hospital of Shanxi Medical University, Tongji Shanxi Hospital, Taiyuan, China

**Keywords:** Virtual reality, Augmented reality, Cardiopulmonary resuscitation, Basic life support, Systematic review, Meta-analysis

## Abstract

**Background:**

Virtual reality (VR) and augmented reality (AR) are emerging technologies that can be used for cardiopulmonary resuscitation (CPR) training. Compared to traditional face-to-face training, VR/AR-based training has the potential to reach a wider audience, but there is debate regarding its effectiveness in improving CPR quality. Therefore, we conducted a meta-analysis to assess the effectiveness of VR/AR training compared with face-to-face training.

**Methods:**

We searched PubMed, Embase, Cochrane Library, Web of Science, CINAHL, China National Knowledge Infrastructure, and Wanfang databases from the inception of these databases up until December 1, 2023, for randomized controlled trials (RCTs) comparing VR- and AR-based CPR training to traditional face-to-face training. Cochrane's tool for assessing bias in RCTs was used to assess the methodological quality of the included studies. We pooled the data using a random-effects model with Review Manager 5.4, and assessed publication bias with Stata 11.0.

**Results:**

Nine RCTs (involving 855 participants) were included, of which three were of low risk of bias. Meta-analyses showed no significant differences between VR/AR-based CPR training and face-to-face CPR training in terms of chest compression depth (mean difference [MD], -0.66 mm; 95% confidence interval [CI], -6.34 to 5.02 mm; *P* = 0.82), chest compression rate (MD, 3.60 compressions per minute; 95% CI, -1.21 to 8.41 compressions per minute; *P* = 0.14), overall CPR performance score (standardized mean difference, -0.05; 95% CI, -0.93 to 0.83; *P* = 0.91), as well as the proportion of participants meeting CPR depth criteria (risk ratio [RR], 0.79; 95% CI, 0.53 to 1.18; *P* = 0.26) and rate criteria (RR, 0.99; 95% CI, 0.72 to 1.35; *P* = 0.93). The Egger regression test showed no evidence of publication bias.

**Conclusions:**

Our study showed evidence that VR/AR-based training was as effective as traditional face-to-face CPR training. Nevertheless, there was substantial heterogeneity among the included studies, which reduced confidence in the findings. Future studies need to establish standardized VR/AR-based CPR training protocols, evaluate the cost-effectiveness of this approach, and assess its impact on actual CPR performance in real-life scenarios and patient outcomes.

**Trial registration:**

CRD42023482286.

**Supplementary Information:**

The online version contains supplementary material available at 10.1186/s12909-024-05720-8.

## Background

Sudden cardiac arrest is a primary health problem around the globe and is estimated to account for 15–20% of all natural deaths in adults [1]. Early and efficient cardiopulmonary resuscitation (CPR) could improve the survival rate after cardiac arrest [2, 3]. However, the rate of bystander CPR and the quality of both in-hospital and out-of-hospital CPR are still low [4–7].

Simulation training is an effective method for improving CPR quality, and instructor-guided face-to-face training has long been the standard [8, 9]. Under the guidance of a qualified instructor, practicing CPR on manikins over time can result in proficient performance. However, traditional face-to-face CPR training has several limitations. Firstly, it requires the availability of qualified instructors and training facilities, which can limit accessibility, particularly in remote areas and for the general public [9]. Secondly, evidence suggests that individuals may experience a decline in CPR proficiency within 3 to 6 months after receiving initial training [10, 11]. Consequently, to maintain high-quality CPR performance, it is crucial to increase the frequency of CPR training [12, 13]. However, this can be challenging due to the limited resources and accessibility associated with traditional face-to-face training. Thirdly, traditional training methods may not be sufficiently engaging or interactive for learners, potentially leading to reduced retention and skill acquisition[14].

Virtual reality (VR) and augmented reality (AR) are emerging technologies that are rapidly gaining traction in medical education [15, 16]. VR/AR can provide immersive, interactive, multi-sensory, and realistic learning environments [17]. To date, VR/AR-based CPR training programmes have been developed. Using VR/AR, CPR training can be conducted without an instructor or even a manikin, allowing for training to be available at any time or place [8, 18]. Compared to face-to-face training, VR/AR-based training has the potential to reach a wider audience, particularly with the rapid advancement of technology. It addresses the limitations of traditional methods by offering increased accessibility and flexibility. Additionally, VR/AR-based training can provide an engaging and interactive experience for learners, which may enhance retention and skill acquisition[19, 20]. However, there is still a debate about whether VR/AR-based training is more effective than face-to-face training in improving CPR quality [7, 8, 18, 21, 22]. Therefore, a meta-analysis is needed to draw a definite conclusion. A previous study [23] has systematically reviewed the available literature before October, 2021 on this topic, but they did not conduct a meta-analysis because of the limited number of studies included. In the present study, we conducted a meta-analysis to compare the efficiency between VR/AR training and face-to-face training, aiming to provide evidence on VR/AR use for CPR training.

## Materials and methods

This study was conducted and reported in accordance with the Preferred Reporting Items for Systematic Review and Meta-Analysis (PRISMA) guidelines [24]. The study protocol was registered in PROSPERO (CRD42023482286).

### Search strategy

We searched for randomized controlled trials (RCTs) that investigated the efficacy of VR- or AR-based CPR training compared to traditional face-to-face training. PubMed, Embase, Cochrane Library, Web of Science, CINAHL, as well as two Chinese databases, China National Knowledge Infrastructure and Wanfang database, were searched. In accordance with our registered protocol, a final search was conducted on December 1st, 2023, to identify publications prior to that date using the following search strategy: “(virtual reality OR augmented reality OR VR OR AR) AND (Cardiopulmonary Resuscitation OR Heart Arrest OR Sudden Cardiac Arrest OR Sudden Cardiac Death OR CPR OR basic life support OR chest compression) AND random*”. We imposed no restrictions on language of publication. The detailed search terms for each database are summarized in Supplemental Table 1.

We also searched the WHO International Clinical Trials Registry Platform, the International Standard Randomized Controlled Trials Number registry, and ClinicalTrials.gov to identify potentially relevant unpublished studies [25, 26].

The reference lists of all included studies were also checked for other relevant studies.

### Selection criteria

We included studies if they met the following criteria:

- Population—Participants undergoing adult CPR training, including medical students, medical staff, and laypersons.
- Intervention—Adult CPR training using VR or AR techniques during practice.
- Control—Traditional face-to-face adult CPR training.
- Outcomes—Measures of CPR performance during final examination, including chest compression depth and rate, overall CPR performance scores, and the proportion of participants meeting CPR guideline quality criteria.
- Study design—Parallel-group RCTs.

We excluded studies that (1) involved pediatric or neonatal CPR training; (2) did not use VR/AR during practice in the intervention group; (3) did not receive face-to-face training in the control group; (4) did not report CPR performance outcomes; or (5) were crossover or cluster RCTs, or published as abstracts, editorials, or letters.

### Study selection

Following the removal of duplicate citations, two independent reviewers reviewed the titles and abstracts of the identified articles. Upon passing the first eligibility screening, the full texts of the studies were obtained in order to determine whether they were eligible for inclusion. The discrepancies were discussed and resolved with the assistance of a third author.

### Data extraction

Two reviewers independently extracted data from the articles using a pre-designed data extraction form, and any disagreements were resolved by a third reviewer. The following information was extracted: author, publication year, study location, sample size, medical background of the participants, training techniques used in the intervention and control groups, and outcomes of interest.

### Primary and secondary outcomes

The primary outcome of the present study was the depth and rate of chest compressions during CPR examination, as they are key quality parameters strongly associated with patient outcomes [8, 27]. The secondary outcomes encompassed the overall CPR performance score and the proportion of participants who met the quality criteria specified in the CPR guidelines (i.e., 50 mm to 60 mm of chest compression depth and 100 to 120 compressions per minute) during examination. We defined the overall CPR performance score as a measure calculated using standardized checklists, such as the European Resuscitation Council (ERC) endorsed CPR checklist and the American Heart Association (AHA) adult CPR skills testing checklist, which evaluate various aspects of CPR performance, including the sequence of steps, compression depth and rate, and other critical components.

### Methodological quality assessment

The Cochrane tool for assessing risk of bias in RCTs (RoB 2.0) [28] was used to assess the methodological quality of the included studies. Two reviewers assessed the methodological quality independently, with disagreements being resolved through discussion with a third reviewer. The RoB 2.0 tool assesses bias in a number of domains, such as the bias arising from the randomization process, the bias due to deviations from intended interventions, the bias due to missing outcome data, the bias in measurement of the outcome, and the bias in selection of the reported result. Based on these domains, studies were categorized as low, some concerns, or high risk of bias.

### Statistical analysis

The meta-analysis was conducted using Review Manager 5.4. Risk ratios (RRs) were calculated with 95% confidence intervals (CIs) for dichotomous data. Continuous data that had the same measure unit (i.e., depth and rate of chest compressions) were calculated as mean differences (MDs) with 95% CIs, while continuous data with different measure units, such as the overall CPR performance score, which used various scoring systems, were calculated as standardized mean differences (SMDs) with 95% CIs. Data reported as medians and interquartile ranges were estimated as means and standard deviations (SDs) based on the methods described by Wan et al. [29] In assessing statistical heterogeneity between studies, the I^2^ statistics were used (30%-60%, 50%-90%, and 75%-100% representing moderate, substantial, and considerable heterogeneity). Assuming the existence of variations across individual studies, we pooled the data using a random-effects model. The statistical significance was set to *P* < 0.05. In the case of outcomes with insufficient data (fewer than two studies), we conducted a narrative description rather than a meta-analysis.

Subgroup analyses were conducted for the primary outcome by type of participants (laypersons, medical students, and medical staff), as well as the method used in the intervention group (VR vs AR, with manikin vs without manikin).

To evaluate whether the meta-analyses were robust, we conducted sensitivity analyses for the primary outcome, by including only studies with low risk of bias or excluding those with estimated means and SDs.

For outcomes with three or more studies included in an analysis, we used funnel plots and Egger's regression tests to assess publication bias. These analyses were performed using Stata 11.0.

## Results

### Study selection

**Fig. 1:**
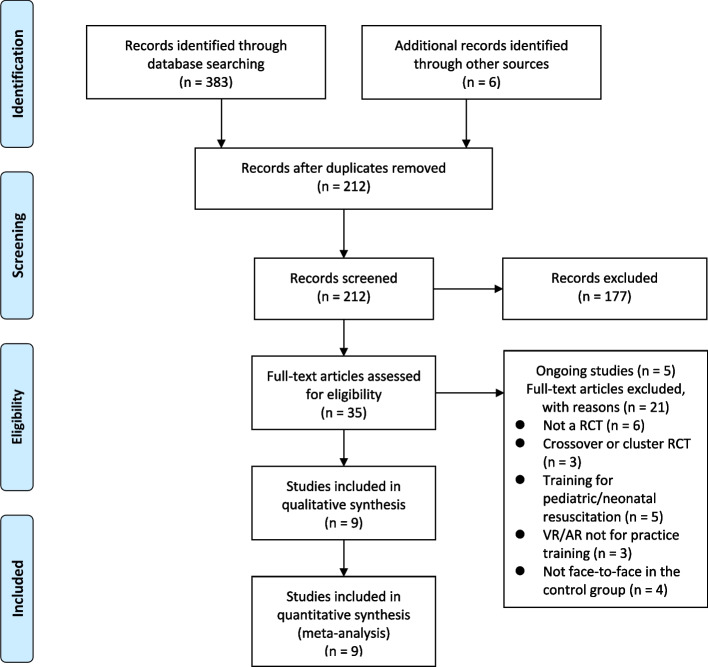
Flowchart of the study selection

Our search strategy identified 389 citations, with 383 obtained through database searching and 6 through other sources. After removing duplicates and screening titles and abstracts, the full texts of 35 studies were obtained in order to determine their eligibility for inclusion. Finally, 9 studies were included in meta-analyses [7, 8, 18, 22, 30–34]. Figure 1 illustrates the study flowchart and selection process. The excluded studies and ongoing studies are summarized in Supplemental Tables 2 and 3.

### Characteristics of the included studies

Nine studies [7, 8, 18, 22, 30–34] involving 855 participants were included in our study. These studies were published between 2020 and 2023, and the sample sizes ranged from 26 to 352. Studies were conducted in China [31, 33, 34], Spain [18, 30], the Netherlands [8], Korea [32], Taiwan [22], and the United Kingdom [7]. Among the studies, seven recruited laypeople [7, 8, 18, 22, 30–32], one recruited medical student [34], and one recruited medical staff [33]. In the intervention group, six studies [7, 8, 18, 22, 32, 34] used VR and three studies [30, 31, 33] used AR during practice training; six studies [7, 22, 30–33] used a manikin during practice training while three studies [8, 18, 34] did not. For all studies included in this review, the control group received face-to-face training with manikins. Table 1 summarizes the characteristics of the included studies.

**Table 1:** Characteristics of the included studies

Author and year	Country/region	Type of participants	Sample size	VR or AR used in the intervention group	Manikin used during VR/AR-guided practice	Outcomes
Alcazar Artero 2023 [18]	Spain	Layperson	64	VR	No	Depth and rate of chest compressions; Proportion of participants meeting CPR guidelines quality criteria
Aranda-Garcia 2023 [30]	Spain	Layperson	60	AR	Yes	Depth and rate of chest compressions
Chang 2023 [22]	Taiwan	Layperson	45	VR	Yes	Overall CPR performance score
Hou 2022 [31]	China	Layperson	27	AR	Yes	Depth and rate of chest compressions; Proportion of participants meeting CPR guidelines quality criteria
Hubail 2022 [7]	UK	Layperson	26	VR	Yes	Depth and rate of chest compressions; Overall CPR performance score
Kim 2023 [32]	Korea	Layperson	121	VR	Yes	Overall CPR performance score
Nas 2020 [8]	Netherlands	Layperson	352	VR	No	Depth and rate of chest compressions; Overall CPR performance score; Proportion of participants meeting CPR guidelines quality criteria
Wu 2022 [33]	China	Medical staff	120	AR	Yes	Overall CPR performance score
Zhou 2022 [34]	China	Medical students	40	VR	No	Depth and rate of chest compressions; Overall CPR performance score

**Fig. 2:**
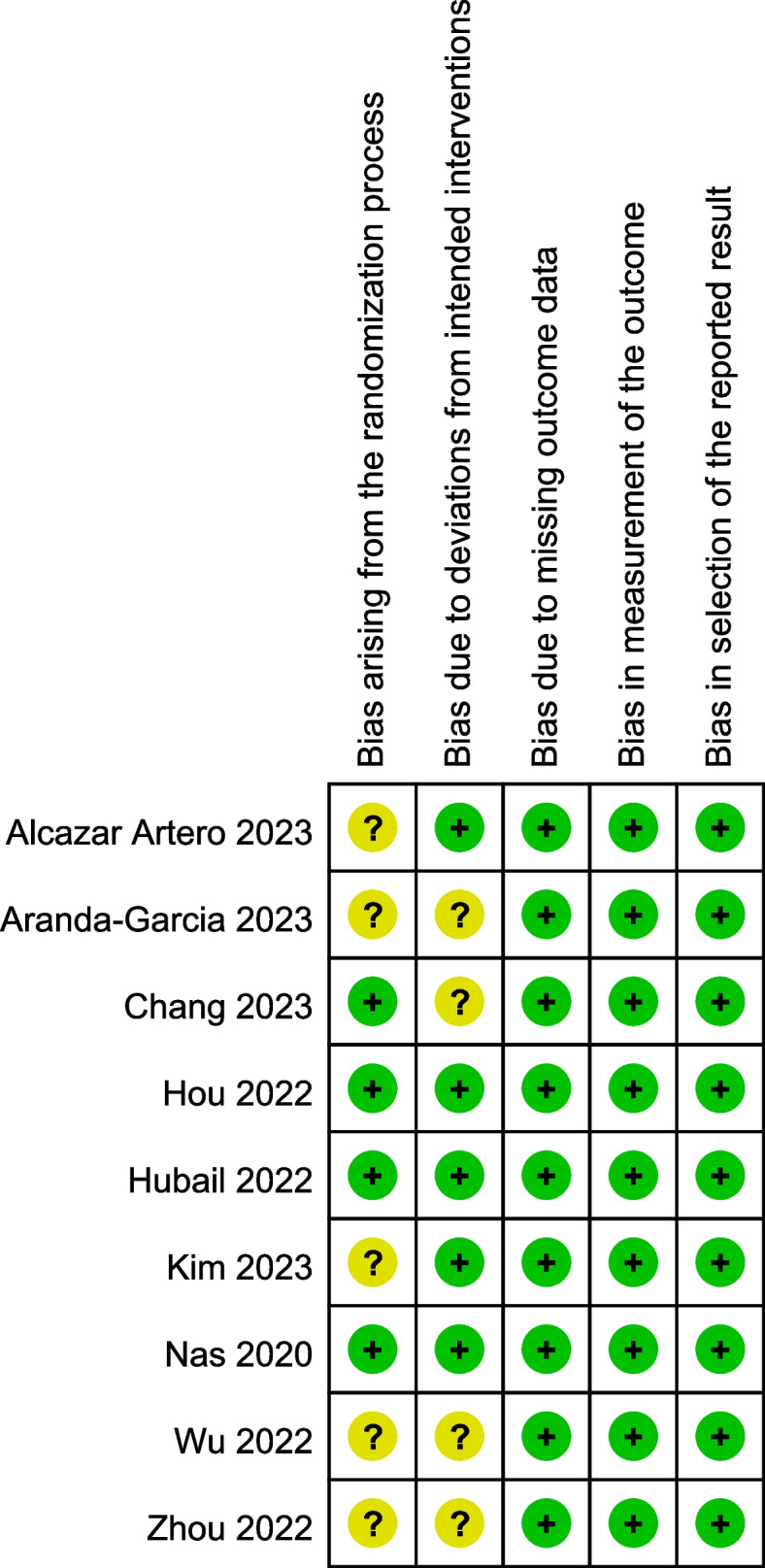
Risk of bias summary. Green represents a low risk of bias, yellow some concerns, and red a high risk of bias

The quality of the included studies was moderate, with three studies [7, 8, 31] found to be at low risk of bias. Figure 2 summarizes the risk of bias assessments.

### Chest compression depth

**Fig. 3:**
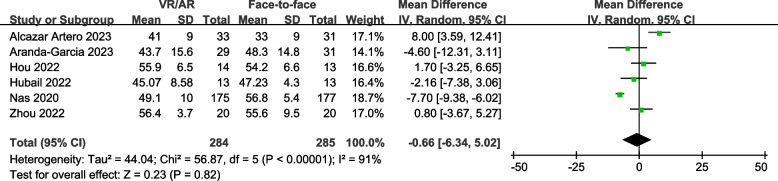
Forest plot for chest compression depth

Six studies [7, 8, 18, 30, 31, 34] (with 569 participants) reported the depth of chest compression during CPR examination. The pooled MD was -0.66 mm (95% CI, -6.34 to 5.02 mm; *P* = 0.82), indicating no significant differences between VR/AR training and face-to-face training (Fig. 3).

Subgroup analyses revealed no significant differences across subgroups. The results from both subgroup and sensitivity analyses were consistent with the primary analysis (Table 2; Supplemental Figs. 1–5).

**Table 2:** Results of subgroup and sensitivity analyses for chest compression depth

	No. of studies	No. of participants	Heterogeneity	Pooled mean difference (95% CI)	Significance	Test for subgroup difference
Type of participants						*P* = 0.67
Layperson	5	529	I^2^ = 92%	-0.95 [-7.66, 5.75]	*P* = 0.78	
Medical students	1	40	-	0.80 [-3.67, 5.27]	*P* = 0.73	
VR or AR used in the intervention group						*P* = 0.95
VR	4	482	I^2^ = 94%	-0.39 [-7.97, 7.19]	*P* = 0.92	
AR	2	87	I^2^ = 45%	-0.73 [-6.74, 5.28]	*P* = 0.81	
Manikin used during VR/AR-guided practice						*P* = 0.83
Yes	3	113	I^2^ = 8%	-0.97 [-4.38, 2.44]	*P* = 0.58	
No	3	456	I^2^ = 96%	0.21 [-9.73, 10.15]	*P* = 0.97	
Sensitivity analysis						-
Overall low risk of bias	3	405	I^2^ = 87%	-3.06 [-9.25, 3.13]	*P* = 0.33	
Data reported as mean and standard deviation	5	509	I^2^ = 93%	0.00 [-6.41, 6.41]	*P* = 1.00	

Furthermore, the Egger's regression test did not exhibit any evidence of publication bias (*P* = 0.097).

### Chest compression rate

**Fig. 4:**
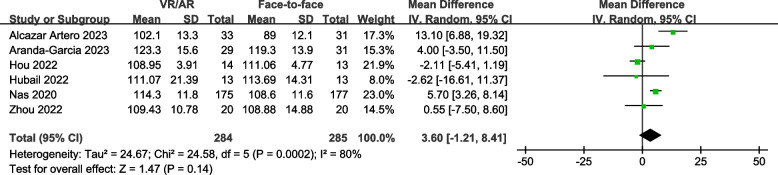
Forest plot for chest compression rate

Six studies [7, 8, 18, 30, 31, 34] (with 569 participants) reported chest compression rates during CPR examination. The pooled MD was 3.60 compressions per minute [95% CI, -1.21 to 8.41 compressions per minute; *P* = 0.14] (Fig. 4), indicating no statistically significant difference between VR/AR training and face-to-face training.

Subgroup analyses revealed a significant difference among subgroups concerning the incorporation of manikins during VR/AR-guided practice (*P* = 0.02), while no statistically significant differences were found in the other subgroups (Table 3; Supplemental Figs. 6–8). VR-based training exhibited higher chest compression rates, whereas no significant association was observed for AR-based training (Supplemental Fig. 7). Additionally, the results showed that chest compression rates were notably higher in scenarios where manikins were not utilized during VR/AR-guided practice (Supplemental Fig. 8).

**Table 3:** Results of subgroup and sensitivity analyses for chest compression rate

	No. of studies	No. of participants	Heterogeneity	Pooled mean difference (95% CI)	Significance	Test for subgroup difference
Type of participants						*P* = 0.47
Layperson	5	529	I^2^ = 83%	4.11 [-1.35, 9.58]	*P* = 0.14	
Medical students	1	40	-	0.55 [-7.50, 8.60]	*P* = 0.89	
VR or AR used in the intervention group						*P* = 0.15
VR	4	482	I^2^ = 64%	5.68 [0.29, 11.07]	*P* = 0.04	
AR	2	87	I^2^ = 53%	-0.02 [-5.70, 5.66]	*P* = 0.99	
Manikin used during VR/AR-guided practice						*P* = 0.02
Yes	3	113	I^2^ = 8%	-0.97 [-4.39, 2.46]	*P* = 0.58	
No	3	456	I^2^ = 70%	6.70 [0.96, 12.44]	*P* = 0.02	
Sensitivity analysis						-
Overall low risk of bias	3	405	I^2^ = 86%	1.19 [-5.54, 7.93]	*P* = 0.73	
Data reported as mean and standard deviation	5	509	I^2^ = 84%	3.50 [-2.08, 9.09]	*P* = 0.22	

Sensitivity analyses yielded consistent results with the primary analysis (Table 3; Supplemental Figs. 9–10). The Egger's regression test demonstrated no evidence of publication bias (*P* = 0.943).

### Overall CPR performance score

The overall CPR performance score during CPR examination was reported in six studies [7, 8, 22, 32–34], involving a total of 828 participants. The studies used various checklists, including the AHA adult CPR skills testing checklist (two studies) [33, 34], the ERC endorsed CPR checklist (one study) [8], the skills testing checklist of the Korea Association of CPR (one study) [32], and the Assessment Checklist adopted from Resuscitation Council UK (one study) [7]. One study [22] did not report the specific checklist used.

**Fig. 5:**
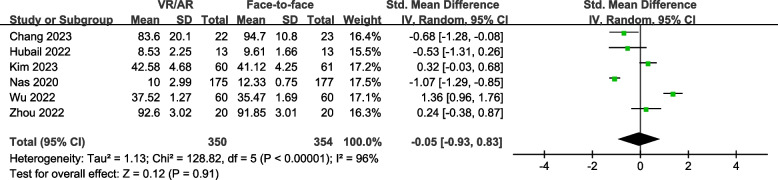
Forest plot for overall CPR performance score

The pooled SMD was -0.05 (95% CI, -0.93 to 0.83; *P* = 0.91) (Fig. 5), suggesting no significant difference between the VR/AR training group and the face-to-face training group. Moreover, the Egger's regression test did not indicate any evidence of publication bias (*P* = 0.409).

### Proportion of participants meeting CPR guidelines criteria

**Fig. 6:**
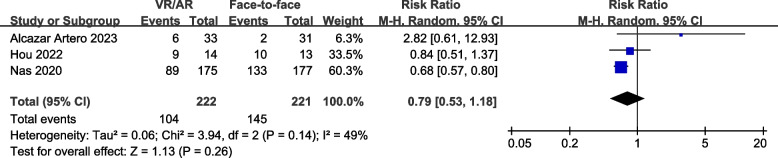
Forest plot for proportion of participants meeting CPR guidelines’ depth criteria

**Fig. 7:**
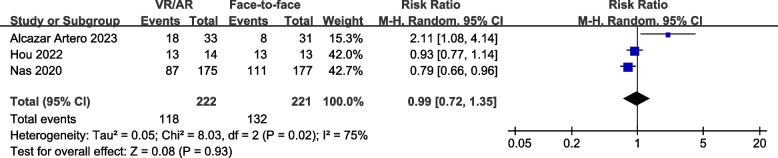
Forest plot for proportion of participants meeting CPR guidelines’ rate criteria

Three studies [8, 18, 31], comprising 443 participants, reported the proportion of participants meeting CPR guidelines criteria. The results indicated no significant differences between VR/AR training and face-to-face training in terms of chest compression depth within the guideline range (RR, 0.79; 95% CI, 0.53 to 1.18; *P* = 0.26) (Fig. 6) and chest compression rate within the guideline range (RR, 0.99; 95% CI, 0.72 to 1.35; *P* = 0.93) (Fig. 7). Additionally, the Egger’s regression test did not reveal any evidence of publication bias (*P* = 0.274 for compression depth; *P* = 0.340 for compression rate).

## Discussion

Our study results indicated that VR/AR-based CPR training yielded similar chest compression depths and rates, comparable CPR performance scores, and an equivalent proportion of participants meeting CPR guidelines when compared to face-to-face training. According to these findings, VR/AR-based training may be as effective as traditional face-to-face training. However, substantial heterogeneity was found among the included studies, which reduced the level of confidence in the findings.

VR/AR technology can facilitate first-person active learning through the creation of immersive and realistic environments [17]. Immersive learning can enhance learning efficiency, as knowledge is retained better when it is directly experienced rather than observed or heard [35]. Studies have demonstrated the effectiveness of VR/AR in facilitating the acquisition of theoretical knowledge, such as anatomy [36]. Furthermore, VR/AR has demonstrated advantages in training skills such as surgery and CPR [18, 37–39]. Incorporating VR/AR into traditional face-to-face CPR training has been found to enhance its effectiveness [22, 40]. Using interactive VR/AR devices, individuals can learn CPR in an automated setting without an instructor or even a manikin [8, 18]. Compared to face-to-face training, this approach would reduce the requirement for qualified instructors and training facilities. The benefits of VR/AR-based training are particularly significant in specific situations, such as the COVID-19 pandemic, when minimizing in-person gatherings is essential and large-scale face-to-face training is not feasible [39].

A growing number of studies have examined the effectiveness of VR/AR technologies for CPR training [8, 39, 41, 42]. Alcazar Artero et al. conducted a systematic review of literature published before October 2021 [23]; however, due to the limited number of RCTs available at that time, they did not perform a meta-analysis. Since then, several RCTs have been published [7, 18, 22, 30–32]. Our study builds on this by incorporating these recent RCTs and conducting a comprehensive meta-analysis to quantitatively compare the effectiveness of VR/AR-based CPR training with traditional face-to-face training. We selected multiple outcome measures to assess the effectiveness of CPR training. Our results indicated that VR/AR-based training led to similar depths and rates of chest compressions as face-to-face training, with sensitivity analysis confirming the robustness of this finding. Additionally, our results showed that VR/AR-based CPR training yielded comparable performance scores and an equivalent proportion of participants meeting CPR guidelines. These findings are consistent with Alcazar Artero et al.'s conclusion that VR/AR-based CPR training could be an effective alternative to traditional methods [23]. However, our meta-analysis provides more robust evidence by quantitatively synthesizing the results of available RCTs.

There was substantial heterogeneity among the included studies. According to the results of subgroup analyses, this could be attributed largely to the training method in the VR/AR group, for example, using manikins or not. The heterogeneity in the VR/AR-based training could also be attributed to other factors, such as the different devices, platforms, and software used. Another potential source of heterogeneity in the meta-analysis could be the variation in assessment checklists for CPR performance among the included studies. These checklists differ in their emphasis on specific aspects of CPR performance and the allocation of points to various components. Due to the limited number of included studies, we were unable to conduct subgroup analyses regarding these factors, nor were we able to identify which VR/AR and training system was most effective for training. We found that VR/AR-based training was more effective in some subgroups, but these results are exploratory and should be verified in future studies.

Our results suggest that VR/AR-based training may serve as an alternative method to face-to-face training. This finding may have significant implications for the development of new CPR training patterns, since VR/AR-based training is easily available and can be accessed at any time or location [8, 18]. By providing VR/AR-based training, it may increase the layperson CPR rate and CPR performance quality. However, further research is required to assess the effects of VR/AR-based training on real-life CPR performance and patient outcome.

VR/AR-based training may have several limitations. A significant consideration is the technology's cost. The expense varies widely depending on the device, platform, and software used. High-quality VR/AR systems with advanced features can be expensive, potentially limiting accessibility in resource-limited settings. However, as technology continues to advance, these costs may decrease, making VR/AR-based training more accessible in the future. Future studies should evaluate the cost-effectiveness of VR/AR-based CPR training.

The acceptance and usability of VR/AR technology are additional concerns. Side effects such as dizziness, blurred vision, and headaches associated with VR/AR use [43] are typically temporary and can be alleviated by breaks; however, these effects may impact training effectiveness and user comfort. Furthermore, inexperienced users may require more time and effort to adapt, potentially hindering initial training efficiency and outcomes. A significant limitation of VR/AR-based training is the lack of haptic feedback, particularly notable in the absence of a manikin, which may compromise learners' ability to accurately perform chest compressions and other essential hands-on CPR skills. To overcome this challenge, future research should investigate the integration of haptic feedback into VR/AR systems [44]. Additionally, variability in VR/AR content and instructional design can impact the quality and effectiveness of training. Therefore, one of the primary objectives for future research is to standardize VR/AR training protocols and ensure high-quality instructional content to maximize the benefits of these technologies.

### Strengths and limitations

Our meta-analysis has several strengths. First, to our knowledge, this is the first comprehensive meta-analysis that thoroughly compares the efficacy of VR/AR-based CPR training with face-to-face CPR training. Second, we performed prespecified subgroup analyses to explore potential sources of heterogeneity and conducted sensitivity analyses to evaluate the robustness of our findings.

Several limitations should be acknowledged in our study. First, there was substantial statistical heterogeneity among the included studies, possibly due to differences in participant characteristics and training methods. This heterogeneity should be considered when interpreting our findings. Second, our primary objective was to compare VR/AR-based training with face-to-face training, so we did not compare the effectiveness of VR/AR with other training methods, such as video or mobile applications.

## Conclusions

Our study showed evidence that VR/AR-based training was as effective as traditional face-to-face CPR training. Considering the accessibility of VR/AR-based training, this finding may have significant implications for facilitating widespread dissemination of CPR training, potentially increasing the proportion of laypersons trained in CPR and improving the quality of CPR performance in real-life situations. However, there was substantial heterogeneity among the included studies, which reduced confidence in the findings. Future research should establish standardized VR/AR-based CPR training protocols and high-quality instructional content, integrate haptic feedback into VR/AR systems, evaluate the cost-effectiveness of this approach, and assess its impact on actual CPR performance in real-life scenarios and patient outcomes.

### Supplementary Information

Supplementary Material 1.

## Data Availability

The datasets used and/or analysed during the current study are available from the corresponding author on reasonable request.

## Abbreviations

CPR: Cardiopulmonary resuscitation
VR: Virtual reality
AR: Augmented reality
RCT: Randomized controlled trial
ERC: European Resuscitation Council
AHA: American Heart Association
RR: Risk ratio
CI: Confidence interval
MD: Mean difference
SMD: Standardized mean difference
SD: Standard deviation
