# Supplementary material for: Effectiveness of virtual and augmented reality for cardiopulmonary resuscitation training: a systematic review and meta-analysis
Source: BMC Med Educ. 2024 Jul 5;24:730. doi: 10.1186/s12909-024-05720-8 (PMC11227211; doi:10.1186/s12909-024-05720-8)

**Effectiveness of virtual and augmented reality for cardiopulmonary resuscitation training: a systematic review and meta-analysis**

Contents

[Supplemental Table 1. Search strategies. 2](#_Toc167704392)

[Supplemental Table 2. Excluded studies. 4](#_Toc167704393)

[Supplemental Table 3. Ongoing studies. 6](#_Toc167704394)

[Supplemental Fig 1. Forest plot for the outcome of chest compression depth (mm): subgroup analysis by type of participants. 7](#_Toc167704395)

[Supplemental Fig 2. Forest plot for the outcome of chest compression depth (mm): subgroup analysis by using VR or AR in the intervention group. 8](#_Toc167704396)

[Supplemental Fig 3. Forest plot for the outcome of chest compression depth (mm): subgroup analysis by whether using manikins during VR/AR-guided practice. 9](#_Toc167704397)

[Supplemental Fig 4. Forest plot for the outcome of chest compression depth (mm): sensitivity analysis by including only studies with low risk of bias. 10](#_Toc167704398)

[Supplemental Fig 5. Forest plot for the outcome of chest compression depth (mm): sensitivity analysis by excluding studies with estimated means and standard deviations. 11](#_Toc167704399)

[Supplemental Fig 6. Forest plot for the outcome of chest compression rate (compressions per minute): subgroup analysis by type of participants. 12](#_Toc167704400)

[Supplemental Fig 7. Forest plot for the outcome of chest compression rate (compressions per minute): subgroup analysis by using VR or AR in the intervention group. 13](#_Toc167704401)

[Supplemental Fig 8. Forest plot for the outcome of chest compression rate (compressions per minute): subgroup analysis by whether using manikins during VR/AR-guided practice. 14](#_Toc167704402)

[Supplemental Fig 9. Forest plot for the outcome of chest compression rate (compressions per minute): sensitivity analysis by including only studies with low risk of bias. 15](#_Toc167704403)

[Supplemental Fig 10. Forest plot for the outcome of chest compression rate (compressions per minute): sensitivity analysis by excluding studies with estimated means and standard deviations. 16](#_Toc167704404)

## Supplemental Table 1. Search strategies.

| **Pubmed search strategy**  ("virtual reality"[Title/Abstract] OR "augmented reality"[Title/Abstract] OR "VR"[Title/Abstract] OR "AR"[Title/Abstract] OR ("virtual reality"[MeSH Terms] OR "augmented reality"[MeSH Terms])) AND ("cardiopulmonary resuscitation"[Title/Abstract] OR "heart arrest"[Title/Abstract] OR "sudden cardiac arrest"[Title/Abstract] OR "sudden cardiac death"[Title/Abstract] OR "CPR"[Title/Abstract] OR "basic life support"[Title/Abstract] OR "chest compression"[Title/Abstract] OR ("cardiopulmonary resuscitation"[MeSH Terms] OR "heart arrest"[MeSH Terms] OR "death, sudden, cardiac"[MeSH Terms])) AND "random*"[All Fields] |
| --- |
| **CENTRAL (Cochrane Central Register of Controlled Trials) search strategy**  #1 (virtual reality or augmented reality or VR or AR):ti,ab.kw  #2 MeSH descriptor: [Virtual Reality] explode all trees  #3 MeSH descriptor: [Augmented Reality] explode all trees  #4 #1 OR #2 OR #3  #5 (Cardiopulmonary Resuscitation or Heart Arrest or Sudden Cardiac Arrest or Sudden Cardiac Death or CPR or basic life support or chest compression):ti,ab.kw  #6 MeSH descriptor: [Cardiopulmonary Resuscitation] explode all trees  #7 MeSH descriptor: (Death, Sudden, Cardiac] explode all trees  #8 #5 OR #6 OR #7  #9 (random*)  #10 #4 AND #8 AND #9 |
| **EMBASE search strategy**  1. (virtual reality or augmented reality or VR or AR).ab,kw,ti.  2. virtual reality/  3. augmented reality/  4. 1 or 2 or 3  5. (Cardiopulmonary Resuscitation or Heart Arrest or Sudden Cardiac Arrest or Sudden Cardiac Death or CPR or basic life support or chest compression).ab,kw,ti.  6. basic life support/  7. cardiopulmonary arrest/  8. resuscitation/  9. 5 or 6 or 7 or 8  10. "random*".af.  11. 4 and 9 and 10 |
| **Web of Science search strategy**  #1 virtual reality or augmented reality or VR or AR (Topic)  #2 Cardiopulmonary Resuscitation or Heart Arrest or Sudden Cardiac Arrest or Sudden Cardiac Death or CPR or basic life support or chest compression (Topic)  #3 ALL=(random*)  #4 #1 AND #2 AND #3 |
| **CINAHL search strategy**  S1 SU virtual reality or augmented reality or VR or AR  S2 SU Cardiopulmonary Resuscitation or Heart Arrest or Sudden Cardiac Arrest or Sudden Cardiac Death or CPR or basic life support or chest compression  S3 TX random*  S4 S1 AND S2 AND S3 |
| **China National Knowledge Infrastructure search strategy (Chinese)**  #1 VR OR 虚拟现实 OR AR OR 增强现实  #2 心肺复苏 OR 胸外按压  #3 随机  #4 #1 AND #2 AND #3 |
| **Wanfang database search strategy (Chinese)**  #1 VR OR 虚拟现实 OR AR OR 增强现实  #2 心肺复苏 OR 胸外按压  #3 随机  #4 #1 AND #2 AND #3 |

## Supplemental Table 2. Excluded studies.

| **Reason for exclusion** | **Studies** |
| --- | --- |
| Not a RCT (n = 6) | 1. Alcazar Artero PM, Pardo Rios M, Greif R, Ocampo Cervantes AB, Gijon-Nogueron G, Barcala-Furelos R, et al. Efficiency of virtual reality for cardiopulmonary resuscitation training of adult laypersons: A systematic review. Medicine (Baltimore). 2023;102(4):e32736.  2. Bench S, Winter C, Francis G. Use of a Virtual Reality Device for Basic Life Support Training: Prototype Testing and an Exploration of Users' Views and Experience. Simul Healthc. 2019;14(5):287-92.  3. Lee DK, Im CW, Jo YH, Chang T, Song JL, Luu C, et al. Comparison of extended reality and conventional methods of basic life support training: protocol for a multinational, pragmatic, noninferiority, randomised clinical trial (XR BLS trial). Trials. 2021;22(1):946.  4. Nas J, Thannhauser J, Vart P, van Geuns RJ, van Royen N, Bonnes JL, et al. Rationale and design of the Lowlands Saves Lives trial: a randomised trial to compare CPR quality and long-term attitude towards CPR performance between face-to-face and virtual reality training with the Lifesaver VR app. BMJ Open. 2019;9(11):e033648.  5. Oh DK, Kim JS, Ryoo SM, Kim YJ, Kim SM, Hong SI, et al. Augmented-Medication CardioPulmonary Resuscitation (AMCPR) trial: a study protocol for a randomized controlled trial. Clin Exp Emerg Med. 2022;9(4):361-6.  6. Zheng J, Du L, Deng X, Zhang L, Wang J, Chen G. Efficacy of virtual reality techniques in cardiopulmonary resuscitation training: protocol for a meta-analysis of randomised controlled trials and trial sequential analysis. BMJ Open. 2022;12(2):e058827. |
| Crossover or cluster RCT (n = 3) | 1. Beom JH, Kim MJ, You JS, Lee HS, Kim JH, Park YS, et al. Evaluation of the quality of cardiopulmonary resuscitation according to vehicle driving pattern, using a virtual reality ambulance driving system: a prospective, cross-over, randomised study. BMJ Open. 2018;8(9):e023784.  2. Figols Pedrosa M, Barra Perez A, Vidal-Alaball J, Miro-Catalina Q, Forcada Arcarons A. Use of virtual reality compared to the role-playing methodology in basic life support training: a two-arm pilot community-based randomised trial. BMC Med Educ. 2023;23(1):50.  3. Semeraro F, Frisoli A, Loconsole C, Banno F, Tammaro G, Imbriaco G, et al. Motion detection technology as a tool for cardiopulmonary resuscitation (CPR) quality training: a randomised crossover mannequin pilot study. Resuscitation. 2013;84(4):501-7. |
| Training for pediatric/neonatal resuscitation (n = 5) | 1. Jeffers JM, Schreurs BA, Dean JL, Scott B, Canares T, Tackett S, et al. Paediatric chest compression performance improves via novel augmented-reality cardiopulmonary resuscitation feedback system: A mixed-methods pilot study in a simulation-based setting. Resusc Plus. 2022;11:100273.  2. Kleinman K, Hairston T, Smith B, Billings E, Tackett S, Chopra E, et al. Pediatric Chest Compression Improvement Via Augmented Reality Cardiopulmonary Resuscitation Feedback in Community General Emergency Departments: A Mixed-Methods Simulation-Based Pilot Study. J Emerg Med. 2023;64(6):696-708.  3. Sarvan S, Efe E. The effect of neonatal resuscitation training based on a serious game simulation method on nursing students' knowledge, skills, satisfaction and self-confidence levels: A randomized controlled trial. Nurse Educ Today. 2022;111:105298.  4. Tsang KD, Ottow MK, van Heijst AFJ, Antonius TAJ. Electronic Decision Support in the Delivery Room Using Augmented Reality to Improve Newborn Life Support Guideline Adherence: A Randomized Controlled Pilot Study. Simul Healthc. 2022;17(5):293-8.  5. Yang SY, Oh YH. The effects of neonatal resuscitation gamification program using immersive virtual reality: A quasi-experimental study. Nurse Educ Today. 2022;117:105464. |
| VR/AR not for practice training (n = 3) | 1. Akaltan KF, Onder C, Vural C, Orhan K, Akdogan N, Atakan C. The effect of game-based learning on basic life support skills training for undergraduate dental students. J Dent Educ. 2023;87(10):1458-68.  2. Aksoy E. Comparing the Effects on Learning Outcomes of Tablet-Based and Virtual Reality-Based Serious Gaming Modules for Basic Life Support Training: Randomized Trial. JMIR Serious Games. 2019;7(2):e13442.  3. Cerezo Espinosa C, Segura Melgarejo F, Melendreras Ruiz R, Garcia-Collado AJ, Nieto Caballero S, Juguera Rodriguez L, et al. Virtual reality in cardiopulmonary resuscitation training: a randomized trial. Emergencias. 2019;31(1):43-6. |
| Not face to face in the control group (n = 4) | 1. Leary M, McGovern SK, Balian S, Abella BS, Blewer AL. A Pilot Study of CPR Quality Comparing an Augmented Reality Application vs. a Standard Audio-Visual Feedback Manikin. Front Digit Health. 2020;2:1.  2. Leary M, McGovern SK, Chaudhary Z, Patel J, Abella BS, Blewer AL. Comparing bystander response to a sudden cardiac arrest using a virtual reality CPR training mobile app versus a standard CPR training mobile app. Resuscitation. 2019;139:167-73.  3. Liu Q, Tang Q, Wang Y. The effects of pretraining intervention in immersive embodied virtual reality cardiopulmonary resuscitation training. Behaviour & Information Technology. 2021;40(12):1265-77.  4. Moll-Khosrawi P, Falb A, Pinnschmidt H, Zollner C, Issleib M. Virtual reality as a teaching method for resuscitation training in undergraduate first year medical students during COVID-19 pandemic: a randomised controlled trial. BMC Med Educ. 2022;22(1):483. |

## Supplemental Table 3. Ongoing studies.

| **Trial ID** | **Title** |
| --- | --- |
| CTRI/2023/03/050897 | To know the benefits of a computer generated educational training material for learning life saving techniques and checking its uses among entry level college students. |
| ISRCTN57122010 | Comparing virtual reality simulation training for resuscitation after cardiac surgery with traditional classroom training |
| JPRN-UMIN000050798 | Verification of the educational effectiveness of a Smartphone Application for Cardiopulmonary Resuscitation Training Using Augmented Reality Technology |
| KCT0006939 | Effects of virtual reality-based professional resuscitation simulation program on nurses' CPR performance ability, performance confidence, and educational satisfaction Effects of virtual reality-based professional resuscitation simulation program on nurses' CPR performance ability, performance confidence, and educational satisfaction |
| NCT04736888 | Effectiveness of Extended Reality CPR Training Methods |

## Supplemental Fig 1. Forest plot for the outcome of chest compression depth (mm): subgroup analysis by type of participants.


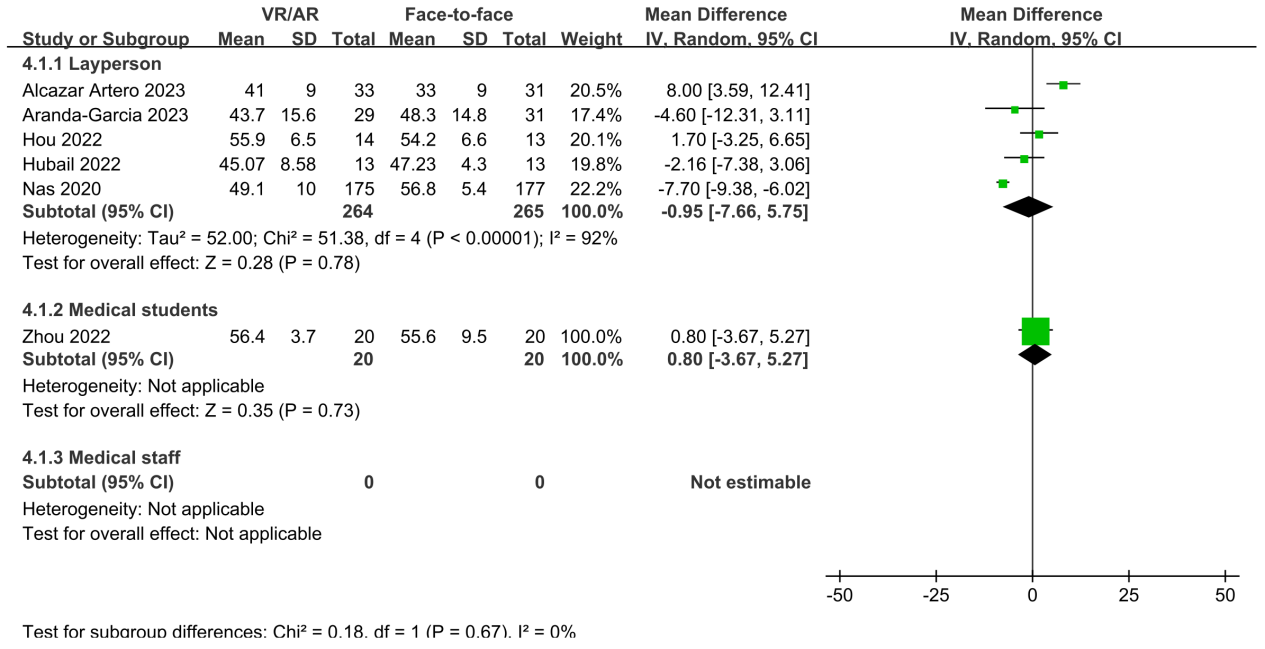


## Supplemental Fig 2. Forest plot for the outcome of chest compression depth (mm): subgroup analysis by using VR or AR in the intervention group.


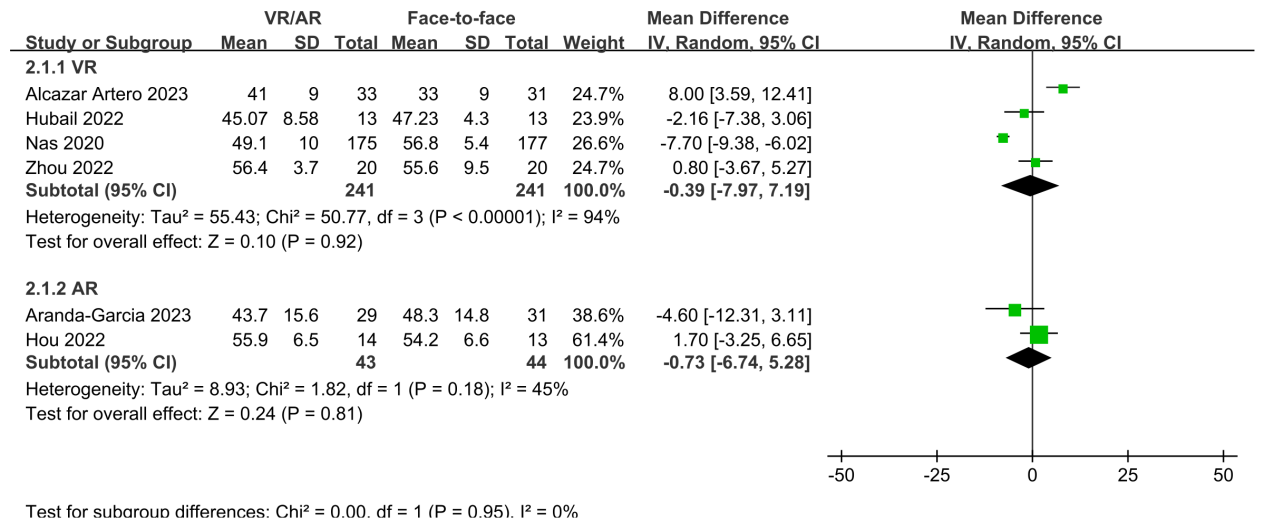


## Supplemental Fig 3. Forest plot for the outcome of chest compression depth (mm): subgroup analysis by whether using manikins during VR/AR-guided practice.


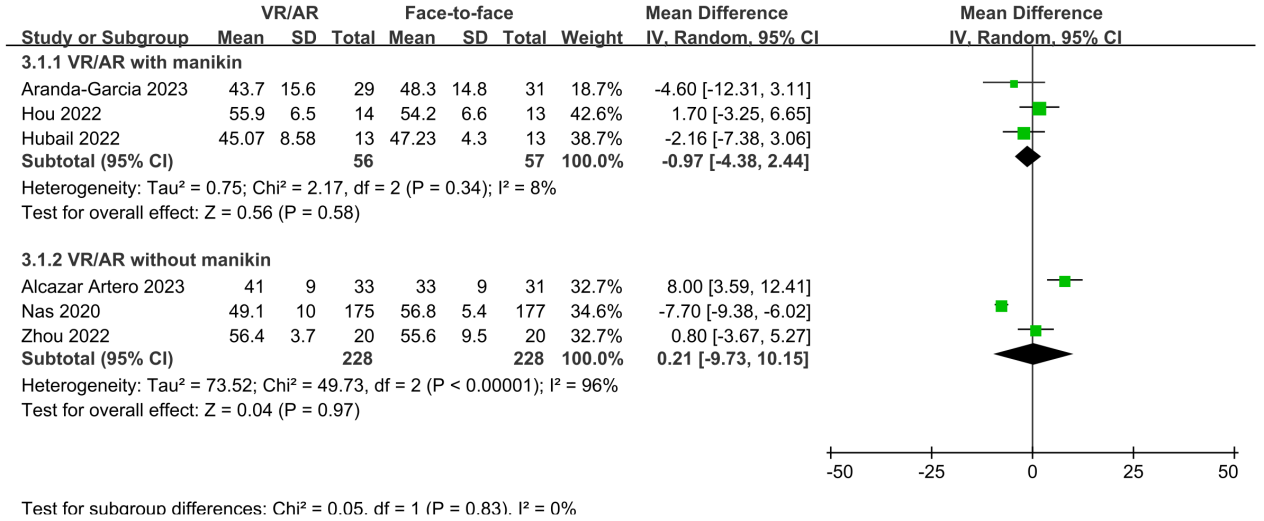


## Supplemental Fig 4. Forest plot for the outcome of chest compression depth (mm): sensitivity analysis by including only studies with low risk of bias.


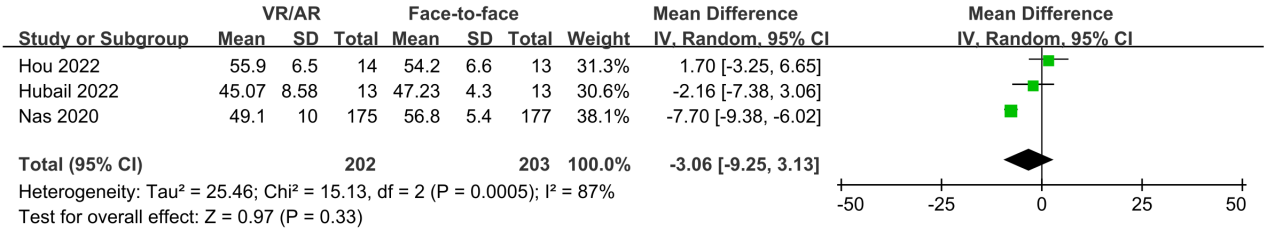


## Supplemental Fig 5. Forest plot for the outcome of chest compression depth (mm): sensitivity analysis by excluding studies with estimated means and standard deviations.


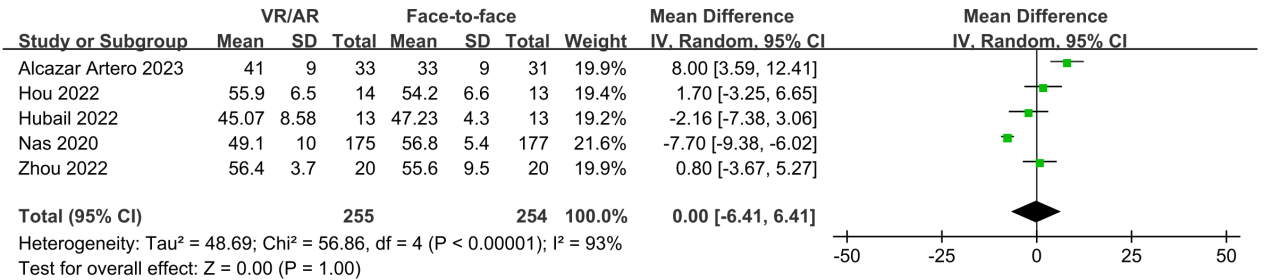


## Supplemental Fig 6. Forest plot for the outcome of chest compression rate (compressions per minute): subgroup analysis by type of participants.


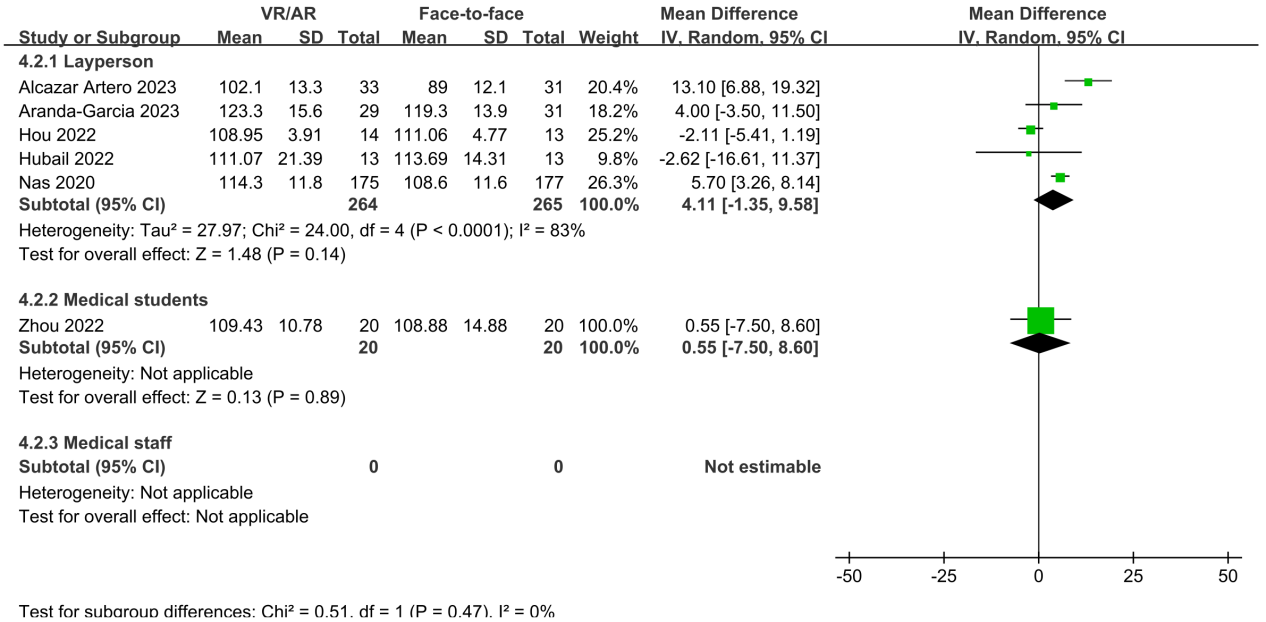


## Supplemental Fig 7. Forest plot for the outcome of chest compression rate (compressions per minute): subgroup analysis by using VR or AR in the intervention group.


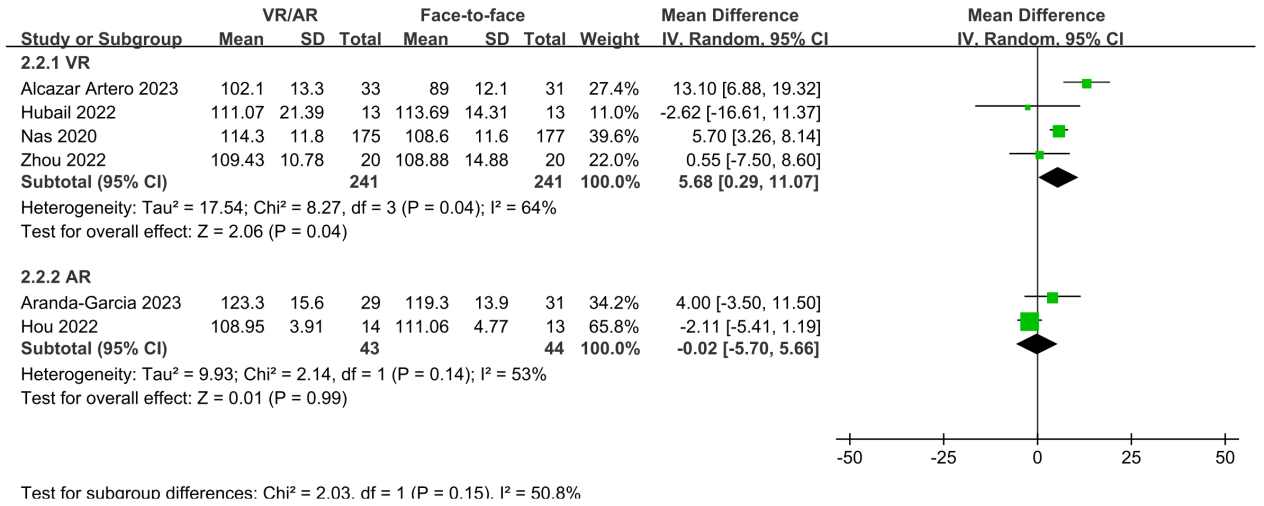


## Supplemental Fig 8. Forest plot for the outcome of chest compression rate (compressions per minute): subgroup analysis by whether using manikins during VR/AR-guided practice.


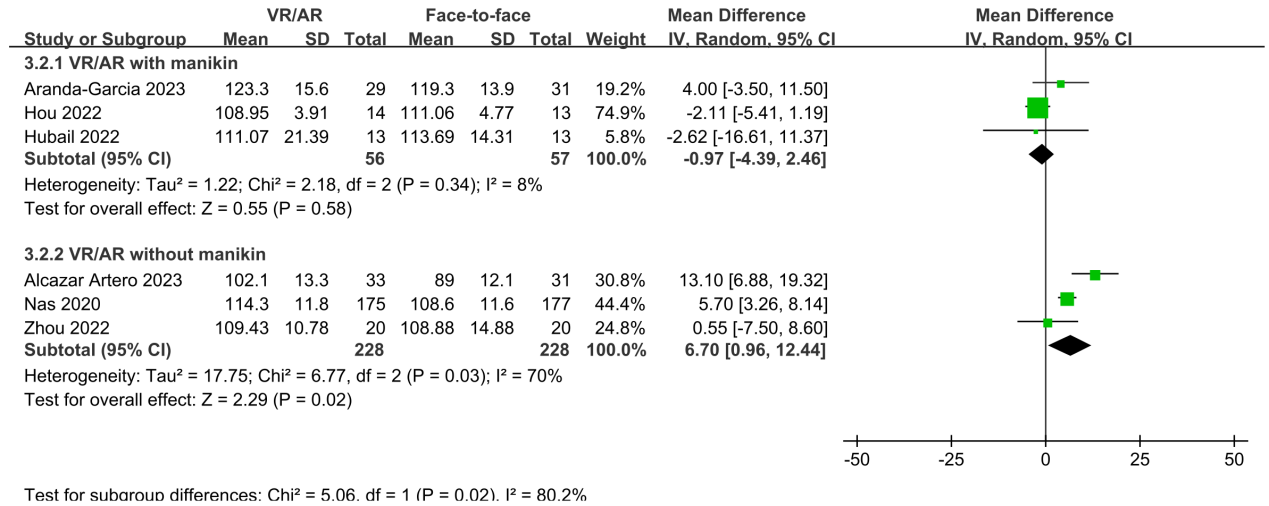


## Supplemental Fig 9. Forest plot for the outcome of chest compression rate (compressions per minute): sensitivity analysis by including only studies with low risk of bias.


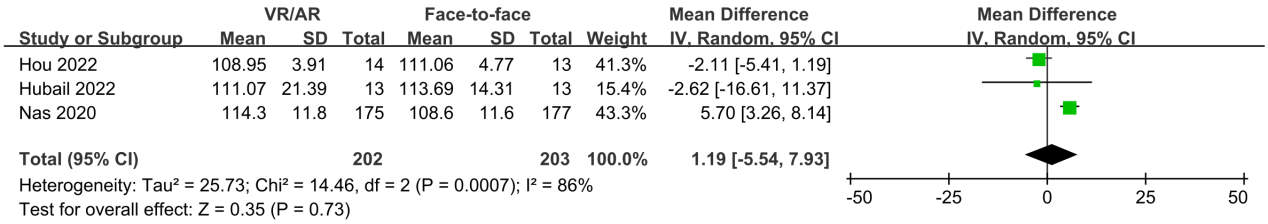


## Supplemental Fig 10. Forest plot for the outcome of chest compression rate (compressions per minute): sensitivity analysis by excluding studies with estimated means and standard deviations.


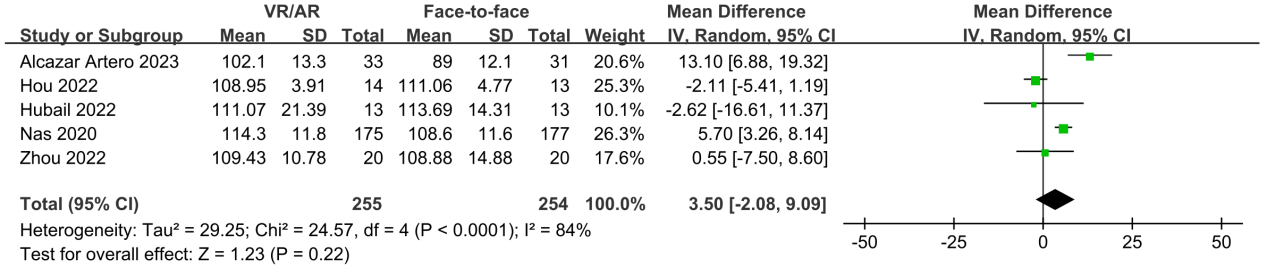

Supplement: Supplementary file 1 — Supplementary Material 1. [file 12909_2024_5720_MOESM1_ESM.docx]
